# Estimation of the adolescent pregnancy rate in Thailand 2008–2013: an application of capture-recapture method

**DOI:** 10.1186/s12884-020-2808-3

**Published:** 2020-02-19

**Authors:** Bunyarit Sukrat, Chusak Okascharoen, Sasivimol Rattanasiri, Wichai Aekplakorn, Jiraporn Arunakul, Kittipong Saejeng, Dankmar Böhning, Ammarin Thakkinstian

**Affiliations:** 1 Department of Clinical Epidemiology and Biostatistics, Faculty of Medicine Ramathibodi Hospital, Mahidol University, Bangkok, Thailand; 2 Department of Pediatrics, Faculty of Medicine Ramathibodi Hospital, Mahidol University, 270 RAMA VI Road. Rachathevi, Bangkok, 10400 Thailand; 3 Department of Community Medicine, Faculty of Medicine Ramathibodi Hospital, Mahidol University, Bangkok, Thailand; 4 Bureau of Reproductive Health, Department of Health, Ministry of Public Health, Nonthaburi, Thailand; 5 Southampton Statistical Sciences Research Institute, University of Southampton, Southampton, UK

**Keywords:** Adolescent, Pregnancy rate, Capture-recapture method

## Abstract

**Background:**

Adolescent pregnancy is an important health and social issue that affects both individual and social well-being. However, deriving a national estimate is challenging in a country with multiple incomplete national databases especially the abortion statistics. The objective of this study was to estimate the adolescent pregnancy rates in Thailand using capture-recapture method.

**Methods:**

An application of capture-recapture method was conducted using two cross-sectional databases (i.e., the national birth registration and the Ministry of Public Health standard health databases) and one hospital-based data source from medical record reviews. A 3-sources capture-recapture with log-linear model was applied to estimate adolescent pregnancy rates.

**Results:**

A total number of 741,084, 290,922 and 25,478 records were respectively identified from the birth registrations, standard health databases and hospital-based survey data during 2008 to 2013. The estimated adolescent pregnancy rates /1000 adolescent women (95% confidence intervals (CI)) ranged from 56.3 (49.4, 66.9) to 70.3 (60.3, 76.6). The estimated rates were about 12–31% higher than adolescent birth rates reported by the Thailand Public Health Statistics.

**Conclusions:**

With the capture-recapture method, more accurate adolescent pregnancy rates were estimated. This method should be able to apply to any setting with similar context.

## Background

Adolescent pregnancy is an important health and social issue which affects both individual and social well-being. Pregnancy related conditions are the leading causes of death among young women [1, 2], and also increase risks of preterm delivery, low birth weight and a number of maternal and neonatal complications [3–5]. A large proportion of pregnancies in young women are unintended and pose a risk of unsafe abortions [6]. Furthermore, adolescent pregnancy also increases socio-economic problems in society such as having poor achievement in education, being a single mother, unemployed and living in poverty [7].

Adolescent pregnancy is a global concern, so it was included in the global health agenda for the Millennium Development Goals (MDGs) in the years 2000 to 2015 and into the Sustainable Development Goals (SDGs) in the years 2016 to 2030. Adolescent birth rate, the number of births per 1000 women aged 15–19 years, was the MDGs indicator under Goal 5B, which was aimed to improve maternal health within 2015 [8]. Reducing adolescent birth rate is currently SDGs indicator number 3.7.2, which is used to improve sexual and reproductive health and the social and economic well-being in adolescents [9]. Unfortunately, adolescent birth rate does not represent the total number of adolescent pregnancies if registry data of abortions and stillbirths are incomplete. Estimation of the total number of adolescent pregnancies is reliable only in the countries with complete data on abortion [10–12]. In countries with restrictive abortion law, underreporting is mainly due to the missing data from induced abortions. Some approaches and indirect estimations have been developed [13–15] to estimate the abortion rates in countries with incomplete abortion statistics, although the most appropriate estimation method is still inconclusive.

Estimation of prevalence or incidence of event or disease condition such as adolescent pregnancy, using complete enumeration of all relevant cases is costly and thus rarely possible, particularly where data registry is not well developed. The indirect estimation method by combining multiple sources of information and deleting duplicated cases always has some degree of undercounting, and thus some adjustment is needed. The capture-recapture (CRC) method has been widely used to estimate population size especially in “hard to reach” populations with incomplete registered data [16–19]. This method can take into account the undercounting of disease/condition using the recapture information, i.e., intersection or overlapping sources, to estimate the number of missing cases under proper assumptions. Although Thailand has well established birth registration, this database includes only live births whereas data for abortion, stillbirth, and miscarriage are not included. A more accurate estimate of adolescent pregnancy rate should lead to better situation analysis and strategic planning for policy makers. We therefore applied the CRC technique to indirectly estimate adolescent pregnancy rate using multiple incomplete data sources.

## Methods

An application of CRC method was conducted using three cross-sectional data sources, which were the national birth registrations, the Ministry of Public Health (MOPH) standard health databases, and hospital-based survey data during the years 2008 to 2013. The study was approved after full review by the Committee on Human Rights Related to Research Involving Human Subjects of the Faculty of Medicine Ramathibodi Hospital (ID 12–55-01) and the Department of Health, the Ministry of Public Health (ID 027). All data owners officially granted access to databases. Pregnant women were included in our study if they were aged 15 to 19 years at delivery. The outcomes of interest were live births and non-live births. The live birth was defined as a complete expulsion or extraction of a product of conception from mother after 22 weeks of gestation with sign of evidence of life or breath. The non-live births included miscarriage, induced abortion, stillbirth, and other abnormal pregnancies which were defined as follows: Abortion, which included induced abortion and miscarriage, which was defined as any delivery which occurred before 22 completed weeks of gestation. Stillbirth was defined as fetal death after 22 completed weeks of gestation. Abnormal pregnancy included ectopic pregnancy, molar pregnancy and others.

### Data sources

Three data sources were used to estimate the adolescent pregnancy rate as follows. First, the National Birth Registration (Source1), is operated by the Bureau of Registration Administration (BRA), the Ministry of Interior. The birth registration is compulsory for all live newborns who are Thai citizens and born in Thailand. The second data source was the MOPH Standard Health Databases (Source2), which included the hospital-based data from the hospitals under the Thailand Universal Healthcare Coverage Scheme. A limitation of this data source is it accounted for only about 80% of all hospitals across the country. To overcome shortcomings of Source1 and Source2, we performed nationwide cross-sectional hospital-based survey (Source3) for the last data source. Pregnancy data of 1321 hospitals providing obstetrics and gynecology services during January 1st, 2008 to December 31st, 2013 were retrieved. A sample size estimation of hospital-based survey was calculated based on estimation of prevalence. This yielded estimated sample size of 29,213 cases. A stratified cluster random sampling without replacement was applied to randomly select sample hospitals across the country. Region and province were considered as stratum and cluster, respectively. All data collection processes were managed by the Data Management Unit (DMU) at the Section of Clinical Epidemiology & Biostatistics, Faculty of Medicine Ramathibodi Hospital, Mahidol University.

### Data management

Data were checked according to year of delivery and age at delivery. Any observation was excluded from databases with the following criteria: duplicated pregnancy of the same person and episodes, which were defined as the pregnancy of the same person whose gestational age intervals were less than 24 weeks from previous gestation. Complying with data privacy regulation, the personal identifiable data in all of the three data sources were deidentified with encryption using message-digest algorithm 5 (MD5). The encrypted Citizen Identification Number (CID) combined with date of delivery were used as a unique key for merging the three databases.

### Statistical analysis

Numbers of pregnant women were described according to data sources and year of delivery. A proportional Venn diagram of the three data sources and the contingency data according to data sources and year of delivery was constructed. To perform CRC analysis, only data from public hospitals under the Office of Permanent Secretary (OPS) were selected from Source1, Source2, and Source3 based on probability of pregnant women being identified from each data source. Pregnancy records were then stratified into live birth and non-live birth groups according to pregnancy outcomes. The pregnant women with multiple gestations were counted as one per one pregnancy episode. In cases of multiple gestations with mixed birth outcomes (live birth plus stillbirth) the pregnant women were only categorized into the non-live birth group to avoid repeated count.

For live-birth group, a CRC was performed using all three data sources. These data were prepared as aggregated data of number of pregnancies in a 2x2x2x6 contingency table. The first three variables referred to data Source1 (Yes/No), Source2 (Yes/No), and Source3 (Yes/No) whereas the last variable referred to year from 2008 to 2013. A CRC was performed using a Poison regression with log link function. The regression models were constructed based on combination of main effects and two-way interaction between each of the data sources. Year of delivery and the interactions between year of delivery and data sources were also put in the models. Performance of each model was assessed and compared using Akaike Information Criterion (AIC) and Bayesian Information Criterion (BIC). The parsimonious model was then used to predict missing numbers of pregnant women who were not identified from Source1, Source2, and Source3. The total number of pregnant women was further calculated by combining the predicted numbers with the total observed number of pregnancies.

For non-live birth, only the data from Source2 and Source3 were used because non-live birth had no chance to appear in the Source1. Therefore, the 2-source CRC was performed to estimate the missing cases and thus the total number of non-live birth pregnancies was filled in.

Adolescence pregnancy rate was estimated by dividing the combined estimated total number of pregnant women from group 1 and group 2 with the number of midyear women population aged 15–19 years, which was annually reported by BPS in Thailand public health statistics [20]. All statistical analyses were performed using STATA version 14.0 [21].

## Results

**Fig. 1:**
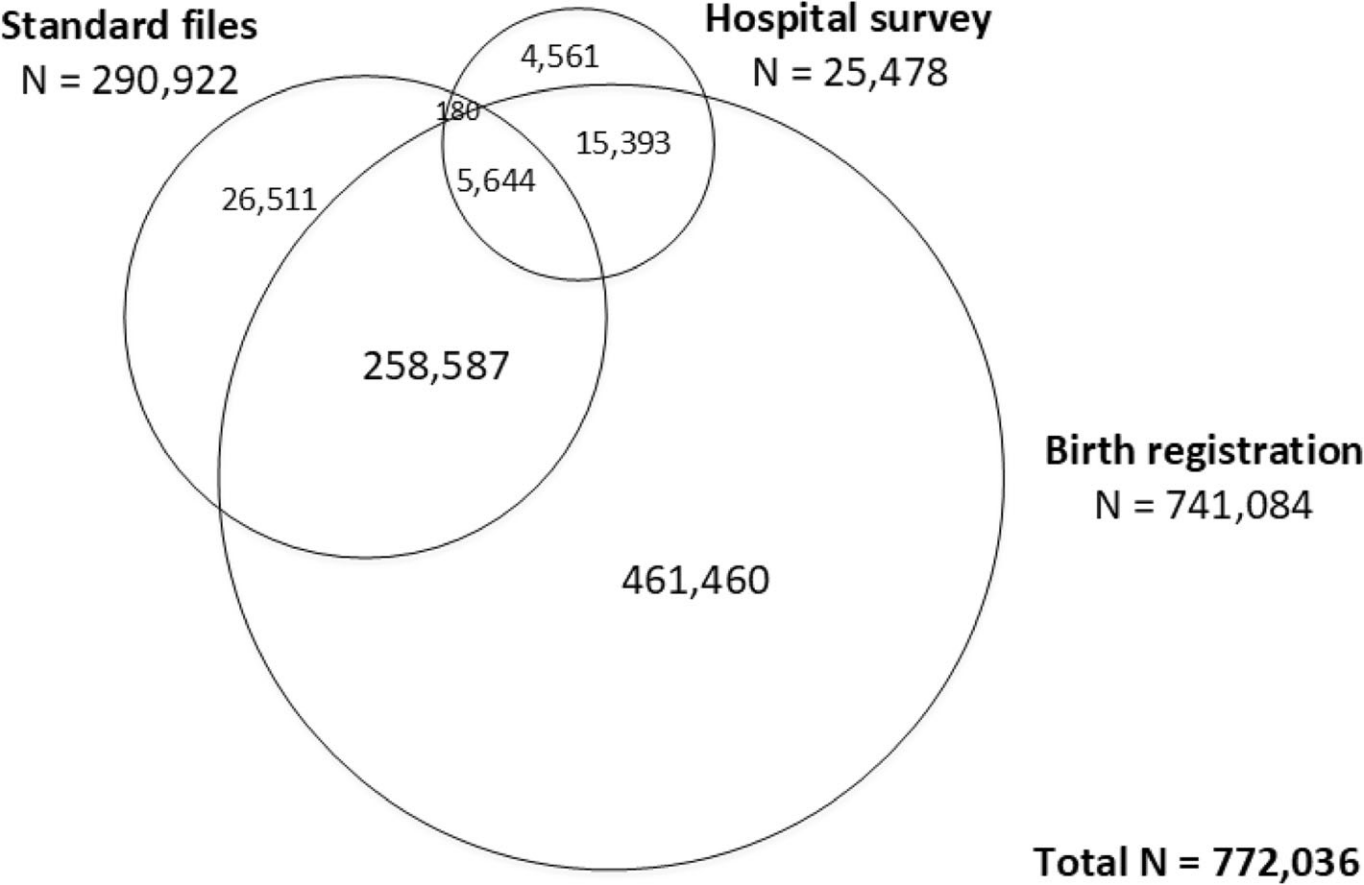
Overall numbers of pregnancies from individual and overlapped data sources

Total numbers of 741,084, 290,922 and 25,478 records from Source1, Source2, and Source3 were respectively eligible yielding 772,036 pregnancy records for further data analysis, see Fig. 1 and Additional file 1 (Fig. A1-A4).

Among them, 122,292 (15.8%) episodes were excluded due to non-OPS hospitals leaving a total of 649,744 episodes of OPS hospitals for CRC consisting of 627,453 and 22,291 pregnant episodes of live-birth (group 1) and non-live birth (group 2), respectively. Numbers of still birth, miscarriage, induced abortions, and abnormal pregnancies are described in Additional file 1-Table A5. Distributions of data were described by sources and time for live birth (group1, Table 1) and non-live birth (group 2, Table 2).

**Table 1:** Data from hospitals under OPS with live birth outcome

Data source			Year						Total
S1	S2	S3	2008	2009	2010	2011	2012	2013	
1	1	1	316	556	828	1342	1295	654	4991
1	1	0	18,339	28,720	40,866	57,369	61,125	38,335	244,754
1	0	1	1199	1587	1623	1461	1546	1862	9278
1	0	0	78,364	67,377	58,257	48,111	45,278	61,980	359,367
0	1	1	4	11	6	4	24	1	50
0	1	0	362	658	1118	1937	2467	1061	7603
0	0	1	207	234	196	238	268	267	1410
0	0	0	–	–	–	–	–	–	–
Total			98,791	99,143	102,894	110,462	112,003	104,160	627,453

S1 denotes Birth Registration

S2 denotes MOPH Standard Health Databases

S3 denotes Hospital-based Survey

**Table 2:** Data from hospitals under OPS with non-live birth outcome

Data source		Year						Total
S2	S3	2008	2009	2010	2011	2012	2013	
1	1	5	55	50	128	49	25	312
1	0	1120	2339	3222	4167	6219	3852	20,919
0	1	175	191	205	184	154	151	1060
0	0	–	–	–	–	–	–	–
Total		1300	2585	3477	4479	6422	4028	22,291

S2 denotes MOPH Standard Health Databases

S3 denotes Hospital-based Survey

For group 1, the best model contained all possible two-way interactions with the AIC and BIC of 596.7 and 643.6, respectively, see Table 3. The missing numbers of pregnancies ranged from 25,819 to 30,218 given the observed numbers of live births of 98,791 to 112,003. The live birth rates were further estimated, which ranged from 52.7 to 59.2 per 1000 adolescent women, see Table 4.

**Table 3:** Model selection

Model	N	Log likelihood	Log likelihood	df	AIC	BIC
		(null model)	(model)			
CRC using record from OPS hospitals with livebirth outcome (group 1)						
S1 S2 S3	42	− 710,020.1	− 1735.5	24	3519.1	3560.8
S1 S2 S3 S2#S3	42	− 710,020.1	− 1379.3	25	2808.6	2852.0
S1 S2 S3 S1#S3	42	− 710,020.1	− 1082.7	25	2215.3	2258.8
S1 S2 S3 S1#S2	42	− 710,020.1	− 509.2	25	1068.3	1111.7
S1 S2 S3 S1#S2 S1#S3	42	− 710,020.1	− 437.5	26	927.0	972.2
S1 S2 S3 S1#S2 S2#S3	42	−710,020.1	− 320.2	26	692.4	737.6
S1 S2 S3 S1#S3 S2#S3	42	−710,020.1	−720.4	26	1492.8	1538.0
S1 S2 S3 S1#S2 S1#S3 S2#S3	42	− 710,020.1	− 271.4	27	596.7	643.6

year year#S1 year#S2 year#S3 were put in all models

S1 denotes Birth Registration

S2 denotes MOPH Standard Health Databases

S3 denotes Hospital-based Survey

N denotes number of observations in the model

# denotes Interaction term

**Table 4:** Estimated results from CRC using model

Year	Observed	Predicted	95% CI	Estimated	95% CI	Number of	Adolescent	95% CI
	count	S000		count		population at midyear	Pregnancy rate	
Group 1: live birth outcome								
2008	98,791	29,315	9996, 48,635	128,106	108,787, 147,426	2,371,583	54.0	45.9, 62.2
2009	99,143	26,911	10,556, 43,266	126,054	109,699, 142,409	2,390,695	52.7	45.9, 59.6
2010	102,894	25,819	9987, 41,652	128,713	112,881, 144,546	2,399,446	53.6	47.0, 60.2
2011	110,462	26,568	10,458, 42,678	137,030	120,920, 153,140	2,413,063	56.8	50.1, 63.5
2012	112,003	30,218	11,788, 48,648	142,221	123,791, 160,651	2,404,152	59.2	51.5, 66.8
2013	104,160	28,875	11,427, 46,323	133,035	115,587, 150,483	2,380,944	55.9	48.5, 63.2
Group 2: non-livebirth outcomes								
2008	1300	4145	961, 7328	5445	2261, 8628	2,371,583	2.3	1.0, 3.6
2009	2585	8242	2513, 13,971	10,827	5098, 16,556	2,390,695	4.5	2.1, 6.9
2010	3477	11,086	3482, 18,689	14,563	6959, 22,166	2,399,446	6.1	2.9, 9.2
2011	4479	14,281	4770, 23,791	18,760	9249, 28,270	2,413,063	7.8	3.8, 11.7
2012	6422	20,475	6122, 34,829	26,897	12,544, 41,251	2,404,152	11.2	5.2, 17.2
2013	4028	12,843	4090, 21,595	16,871	8118, 25,623	2,380,944	7.1	3.4, 10.8
Total estimate: Group 1 + Group 2								
2008	100,091	33,460	10,957, 55,964	133,551	111,048, 156,055	2,371,583	56.3	46.8, 65.8
2009	101,728	35,153	13,069, 57,236	136,881	114,797, 158,964	2,390,695	57.3	48.0, 66.5
2010	106,371	36,905	13,469, 60,341	143,276	119,840, 166,712	2,399,446	59.7	49.9, 69.5
2011	114,941	40,849	15,228, 66,469	155,790	130,169, 181,410	2,413,063	64.6	53.9, 75.2
2012	118,425	50,694	17,910, 83,477	169,119	136,335, 201,902	2,404,152	70.3	56.7, 84.0
2013	108,188	41,718	15,517, 67,918	149,906	123,705, 176,106	2,380,944	63.0	52.0, 74.0

**Fig. 2:**
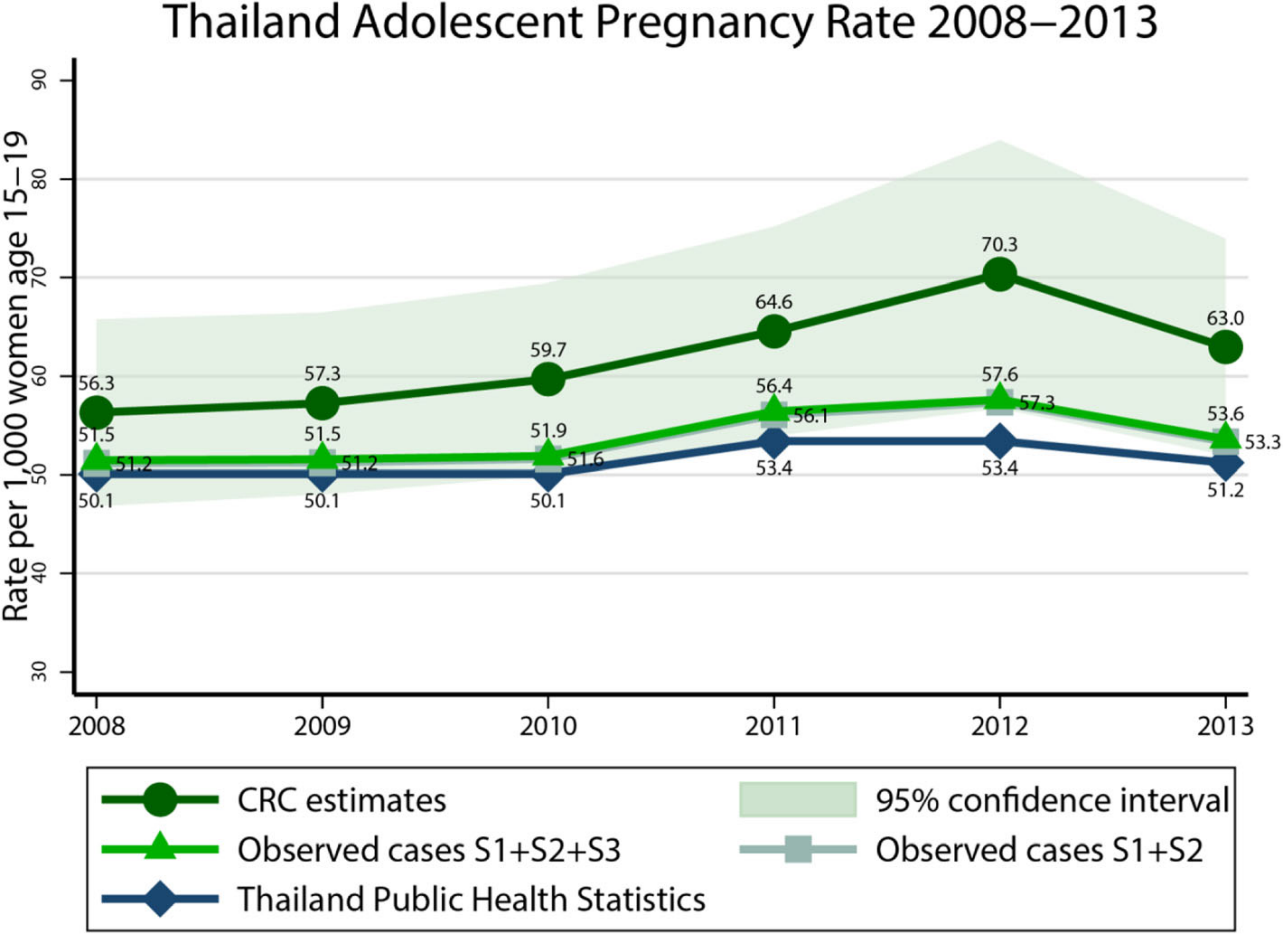
Comparison of adolescent pregnancy rates estimated by three methods

For non-live births, a total of 22,291 observations from only Source2 and Source3 were used for CRC analysis, see Table 2. The estimated total number of non-live births ranged from 5445 to 26,897 with pregnancy rates of 2.3 to 11.2, see Table 4. Finally, the total count of numbers of non-live births were then combined with numbers of live births, yielding a total number of pregnancies of 133,551 to 169,119, which gained about 5445 to 26,898 more pregnancies compared with estimated numbers by live birth only. The adolescent pregnancy rate trended to increase significantly from 56.3 to 70.3 during the years from 2008 to 2012 (Chi-square for trend = 3.54, *p* = 0.009), but it decreased to 63.0 in 2013, see Table 4. Adolescent pregnancy rates were estimated by CRC and actual observed data were compared indicating higher estimated rates by CRC than Source1 only (adolescent birth rate), Source1 plus Source2, and Source1 plus Source2 plus Source3 with the corresponding case detection rates of 75.9–89.0%, 81.5–90.8% and 81.9–91.4%, respectively, see Fig. 2.

## Discussion

This study was conducted applying CRC analysis to estimate adolescent pregnancy rate in Thailand using a log-linear model approach which indicated a significant trend of increasing rate during the years 2008 to 2012, but declining in the year 2013. In addition, the estimated rates were higher in CRC method than the actual observed data by the Public Health Statistics. Estimation of adolescent pregnancy rate is still challenging in many countries, particularly where abortion is still restricted and thus only minimal cases estimations were mostly reported [22, 23].

Although birth registration in Thailand covers as high as 99% of all births [24], data for abortion, stillbirth, and miscarriage are incompletely registered with some degree of underreports. Applying CRC with a log-linear model for estimation of these numbers yielded many advantages as follows: first, all models were constructed under unified statistical framework, and model selection criteria were available for comparing models; second, dependence of data sources could be incorporated by adding interactions between each pair of data sources; and third, the covariates could be taken into account by adding in the model; and all inferences are within the statistical framework [17, 19].

However, the following limitations which might violate assumptions for performing CRC, were difficult to avoid [17, 19, 25]. First limitation was about the assumption that study population should be in closed system during the study period. Although we studied only subjects with Thai nationality, immigration still occurred and could not be avoided. The second limitation was from the assumption which stated individual subjects should be matched from capture to recapture. This refers to the correctness of the identification of subjects and matching them between different data sources, so each individual subject has positive probability to be ascertained by any data source, i.e., missing from any data source should not be a ‘structural zero’ or missing due to impossibility [19]. We were strongly concerned about this issue and performed two steps of CRC, i.e., predicted numbers of live births using three sources of data and non-live births using only Source2 and Source3. Only data from OPS hospitals were selected to keep the probability of pregnant women being identified from each data source to not be zero. The third limitation was from assumption concerning independence of data sources. Source independence can be accounted for by adding the interaction between the pair of data sources into the models. However, the highest order interaction must be assumed to be zero to allow identifiability, which could not be avoided for 2-source CRC in non-live birth group.

The fourth limitation was from assumption about capture homogeneity, which states each individual has the same chance to be ascertained by each data source. Heterogeneity among individuals may induce sources of dependence which can be partially reduced by stratified analysis. The fifth limitation was early pregnancy loss which would not require hospitalization and so could not be included in the samples and thus the estimation procedure.

Our CRC estimates yielded higher adolescent pregnancy rates than estimates based on actually observed data sources, particularly in non-live birth which was the consequence of adding Source2 to Source1. Therefore, we encourage applying CRC to provide more accurate estimation of adolescence pregnancy rate particularly in countries with restrictive abortion law. This will lead health care providers and policy makers to allocate resources properly. However, Source1 and Source2 are needed to improve the quality of the data, especially the identification using CID. The hospital-based survey should be performed regularly depending on feasibility and available funding and applying CRC method to provide more accurate estimation. For the non-livebirth group, the two-source CRC analysis has many theoretical limitations, so the third or fourth data sources should be sought to improve performances of CRC analysis and thus provide more valid results.

## Conclusion

CRC method indicated that estimated adolescent pregnancy rates were much higher than the adolescent birth rates reported in Public Health Statistics. These two indicators should be used altogether for country situation analysis and strategic planning. This method can be applied not only in Thailand, but also other countries with similar contexts.

## Supplementary information

**Additional file 1: Figure A1** Data management flow. **Figure A2** Data management flow for Birth Registration Database. **Figure A3** Data management flow for Standard Health Databases. **Figure A4** Data management flow for Hospital-based Survey. **Table A5** Pregnancy outcomes by data sources.

## Data Availability

The datasets used and/or analysed during the current study are available from the corresponding author on reasonable request.

## Abbreviations

AIC: Akaike Information Criterion
BIC: Bayesian Information Criterion
CID: Citizen identification number
CRC: Capture-recapture
MD5: Message-digest algorithm 5
MDGs: Millennium Development Goals
MOPH: Ministry of Public Health
OPS: Office of Permanent Secretary
SDGs: Sustainable Development Goals
Source1: Birth Registration Database
Source2: Standard Health Databases
Source3: Hospital-based Survey
