# Supplementary material for: Estimation of the adolescent pregnancy rate in Thailand 2008–2013: an application of capture-recapture method
Source: BMC Pregnancy Childbirth. 2020 Feb 19;20:120. doi: 10.1186/s12884-020-2808-3 (PMC7031918; doi:10.1186/s12884-020-2808-3)
Supplement: Supplementary file 1 — Additional file 1: Figure A1 Data management flow. Figure A2 Data management flow for Birth Registration Database. Figure A3 Data management flow for Standard Health Databases. Figure A4 Data management flow for Hospital-based Survey. Table A5 Pregnancy outcomes by data sources. [file 12884_2020_2808_MOESM1_ESM.docx]

Appendix

1. Figure A1 Data management flow

2. Figure A2 Data management flow for Birth Registration Database

3. Figure A3 Data management flow for Standard Health Databases

4. Figure A4 Data management flow for Hospital-based Survey

5. Table A5 Pregnancy outcomes by data sources

Figure A1 Data management flow


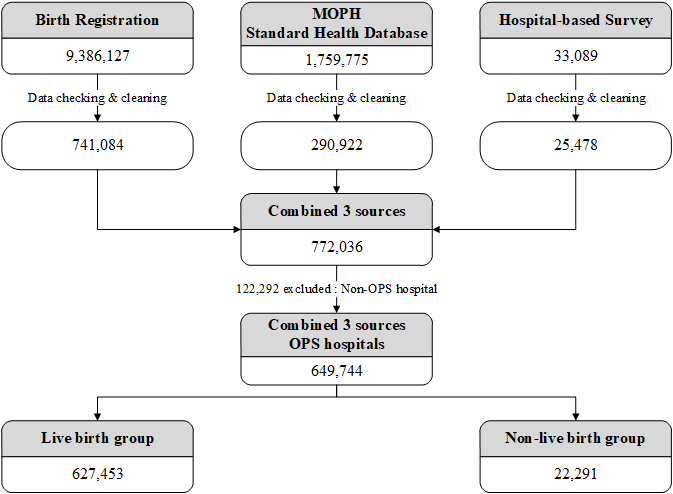


Figure A2 Data management flow for Birth Registration Database


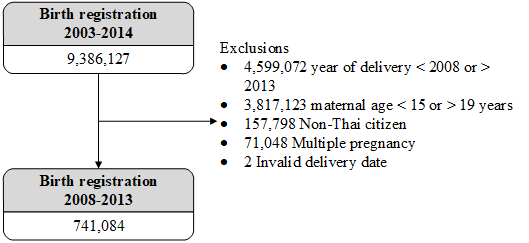


Figure A3 Data management flow for Standard Health Databases


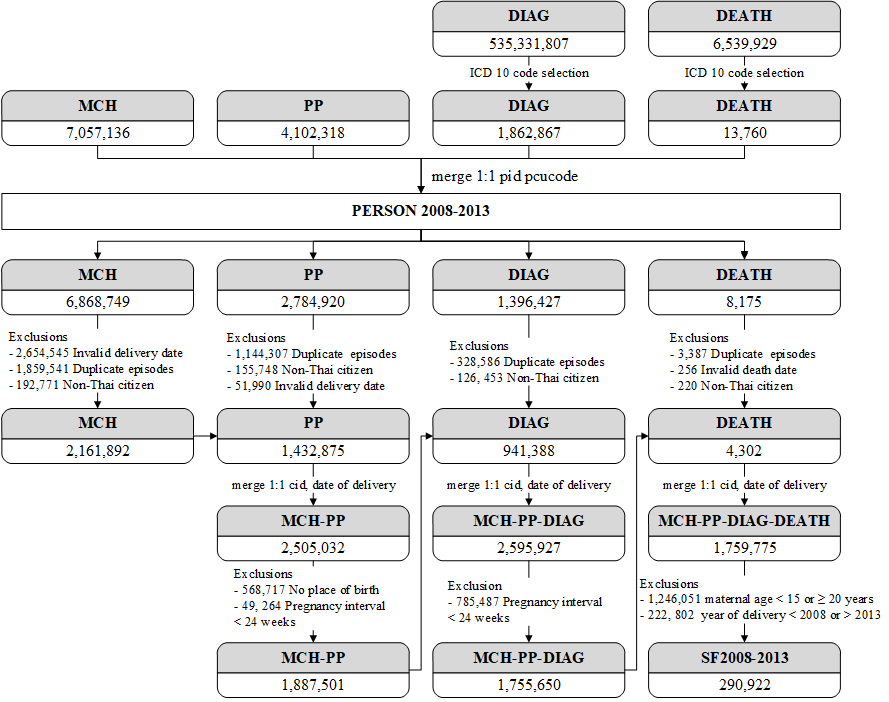


Figure A4 Data management flow for Hospital-based Survey


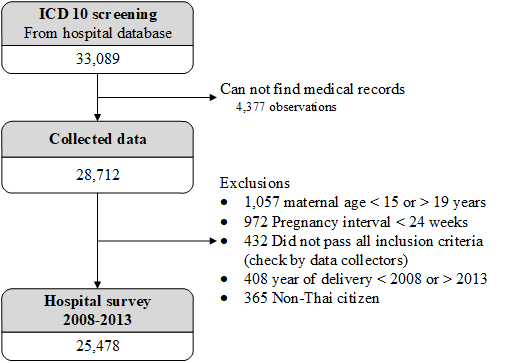


Table A5 Pregnancy outcomes by data sources

| **Pregnancy outcomes** | **Data sources** | | |
| --- | --- | --- | --- |
|  | **Birth Registration (%)** | **Standard Health Databases (%)** | **Hospital-based Survey (%)** |
| Livebirth | 741,084 (100) | 266,007 (91.6) | 23,542 (92.4) |
| Stillbirth |  | 504 (0.2) | 133 (0.5) |
| Miscarriage |  | 10,582 (3.6) | 979 (3.8) |
| Induced abortion |  | 7,471 (2.6) | 350 (1.4) |
| Abnormal pregnancy |  | 5,866 (2.0) | 474 (1.9) |
| Total | 741,084 (100%) | 290,430 (100%) | 25,478 (100%) |
